# Geographical Variability Affects CCHFV Detection by RT–PCR: A Tool for In-Silico Evaluation of Molecular Assays

**DOI:** 10.3390/v11100953

**Published:** 2019-10-16

**Authors:** Cesare E. M. Gruber, Barbara Bartolini, Concetta Castilletti, Ali Mirazimi, Roger Hewson, Iva Christova, Tatjana Avšič, Roland Grunow, Anna Papa, María P. Sánchez-Seco, Marion Koopmans, Giuseppe Ippolito, Maria R. Capobianchi, Chantal B. E. M. Reusken, Antonino Di Caro

**Affiliations:** 1 National Institute for Infectious Diseases (INMI) “L. Spallanzani” IRCCS, WHO Collaborating Center for clinical care, diagnosis, response and training on Highly Infectious Diseases, 00149 Rome, Italy; cesare.gruber@inmi.it (C.E.M.G.); concetta.castilletti@inmi.it (C.C.); giuseppe.ippolito@inmi.it (G.I.); maria.capobianchi@inmi.it (M.R.C.); antonino.dicaro@inmi.it (A.D.C.); 2 Public Health agency of Sweden, 17182 Solna, Sweden; ali.mirazimi@folkhalsomyndigheten.se; 3 National veterinary Institute, 75189 Uppsala, Sweden; 4 Department of laboratory Medicine, Clinical Microbiology, Karolinska Institute and Karolinska, 17177 Stockholm, Sweden; 5 Public Health England, National Infection Service WHO Collaborating Centre for Virus Reference and Research (Special Pathogens) Porton Down, Salisbury SP40JG, UK; Roger.Hewson@hpa.org.uk; 6 National Reference Laboratory on Vector-Borne Pathogens, Leptospira and Listeria, Microbiology Department, National Center of Infectious and Parasitic Diseases, 1504 Sofia, Bulgaria; iva_christova@yahoo.com; 7 Faculty of Medicine, Institute of Microbiology and Immunology, 1000 Ljubljana, Slovenia; Tatjana.Avsic@mf.uni-lj.si; 8 Centre for Biological Threats and Special Pathogens, Highly Pathogenic Microorganisms (ZBS 2), Robert Koch Institute, 13353 Berlin, Germany; GrunowR@rki.de; 9 Department of Microbiology, Medical School, Aristotle University of Thessaloniki, 54124 Thessaloniki, Greece; annap@med.auth.gr; 10 National Centre of Microbiology, Institute of Health “Carlos III”, Majadahonda, 28220 Madrid, Spain; paz.sanchez@isciii.es; 11 Erasmus MC, Department of Viroscience, WHO Collaborating Centre for arbovirus and viral hemorrhagic fever reference and research, 3015 CN Rotterdam, The Netherlands; m.koopmans@erasmusmc.nl (M.K.); c.reusken@erasmusmc.nl (C.B.E.M.R.); 12 Centre for Infectious Disease Control, National Institute for Public Health and the Environment, 3721 MA Bilthoven, The Netherlands

**Keywords:** CCHFV, molecular detection, Crimean–Congo hemorrhagic fever virus, arthropod-borne virus, laboratory preparedness, emerging diseases

## Abstract

The Crimean–Congo hemorrhagic fever virus (CCHFV) is considered to be a major emerging infectious threat, according to the WHO R&D blueprint. A wide range of CCHFV molecular assays have been developed, employing varied primer/probe combinations. The high genetic variability of CCHFV often hampers the efficacy of available molecular tests and can affect their diagnostic potential. Recently, increasing numbers of complete CCHFV genomic sequences have become available, allowing a better appreciation of the genomic evolution of this virus. We summarized the current knowledge on molecular methods and developed a new bioinformatics tool to evaluate the existing assays for CCHFV detection, with a special focus on strains circulating in different geographical areas. Twenty-two molecular methods and 181 sequences of CCHFV were collected, respectively, from PubMed and GenBank databases. Up to 28 mismatches between primers and probes of each assay and CCHFV strains were detected through in-silico PCR analysis. Combinations of up to three molecular methods markedly decreased the number of mismatches within most geographic areas. These results supported the good practice of CCHFV detection of performing more than one assay, aimed for different sequence targets. The choice of the most appropriate tests must take into account patient’s travel history and geographic distribution of the different CCHFV strains.

## 1. Introduction

The Crimean–Congo hemorrhagic fever virus (CCHFV) is a tick-borne, negative-sense, single-stranded RNA orthonairovirus, and the causative agent of the Crimean–Congo hemorrhagic fever (CCHF). The CCHFV is considered to be one of the major emerging infectious threats as it might cause a severe disease, with increasing number of cases of infection in Africa, Middle East, Asia, and parts of Europe [1,2].

Rapid diagnosis is crucial to ensure the implementation of appropriate infection control measures and guide post exposure prophylaxis [3]. Among the diagnostic tests available, reverse-transcriptase PCR (RT–PCR) is the method of choice for rapid laboratory diagnosis during the acute phase of infection [4]. However, the efficacy of the available molecular tests is hampered by the high genetic variability of the virus that displays the greatest degree of sequence diversity of any arbovirus species [4,5].

CCHFV is an enveloped virus with a tripartite [small (S), medium (M), and large (L)] negative-sense single strand RNA genome [6], characterized by a degree of sequence diversity of 20%, 22%, and 31% among the S-, L-, and M-segments of the virus genome [5]. The mechanisms responsible for this marked degree of sequence diversity include genetic drift or separate evolution of lineages in geographically constrained reservoirs. Long-distance transport in infected ticks by migratory birds, or trade in viremic life stock can result in multiple strain variants occurring in the same endemic area. Segment reassortment as a result of co-infection can also increase the diversity of CCHFV strains [4,7,8].

Based on geographical origin and phylogenetic analyses of the S gene segment, CCHFV has been previously classified into six main geographical clades [4]—three predominantly diffused in Africa (Clades I-III) [9,10], two in Europe (Clades V and VI) [11,12,13], and one in Asia (Clade IV) [14,15,16,17].

Most of the isolates causing outbreaks in Eastern Europe belong to Clade V, whereas Clade VI includes largely divergent strains isolated from ticks in Greece (including the strain AP92) [4,18] and Turkey [19]. Isolates belonging to the “African” Clade III were also collected in Spain from infected ticks in 2010 [20] and during 2011–2015 [21], which were found to have caused human infections in 2016 [22]. One more recent human infection in Spain in 2018 were caused by a CCHFV not yet characterized [23].

Moreover, in some cases, different CCHFV geographic clades are characterized by different pathogenic potentials, as in the case of Clade VI in Greece, where, despite high antibody prevalence, very few and mostly mild human cases have been reported [24].

Viral diversity can strongly affect sensitivity of molecular tests when mutations occur at the binding site of primers and probes [25]. In particular, mismatches occurring within the 3’-end can dramatically affect fragment amplification [26,27].

Experimental validation of molecular methods can usually be performed on a limited set of biological samples that might not be representative of the viral population from the different outbreak-prone geographical areas. Nowadays, the increasing number of complete viral sequences that can be retrieved from public databases provide a worthwhile resource for evaluating the genetic variation within viral strains. However, many sequence entries have been assembled using classic RT–PCR approaches and the use of general terminal sequencing primers; as a consequence, published sequence fragment termini often include primer sequences. Very few segments have been assembled using molecular techniques specific for terminal sequencing, such as rapid amplification of cDNA ends (RACE).

Many tools were developed for designing in-silico PCR methods [28,29]. Here, we describe a bioinformatic tool (CCHFV Primer Checker) to evaluate the detection efficacy of the existing molecular assays and to propose specific sets of assays to detect as many CCHFV strains from all geographic regions, as possible.

## 2. Materials and Methods

### 2.1. Data Collection

Molecular methods included in this analysis were obtained by a web-search in PubMed or were directly indicated by the authors. A deliberately relaxed query was done to capture a larger-than-required set of articles. The query included the terms “CCHF”, “CHF”, “CCHFV”, “CHFV”, “Congo virus”, or “Crimean–Congo”; filtered by the possibilities of “molecular diagnostics”, “molecular method”, “molecular testing”, “PCR”, “RT–PCR”, “real time PCR”, “NAT”, “NAAT”, “LAMP”,”TMA”, or “transcription-mediated amplification”.

All CCHFV genomes available by 31 December 2018 were retrieved from the GenBank (https://www.ncbi.nlm.nih.gov/nucleotide/), using “txid1980519[Organism]” as the term of query. All S segments including the complete coding region, with available data about host, collection country, and collection date were selected. The S segment sequence of the CCHFV that caused a case of infection in Spain in 2018 [23] was also added to the analysis, although the sequence was not complete [30].

### 2.2. Phylogenetic Analysis

The analyses was focused on the S segment, as the most conserved segment across the CCHFV clades [5,31], and the main target for molecular assays. CCHFV complete coding sequences of the S segment from the different viral strains were selected and clustered at 100%, with CD–HIT v4.6; all sequences were then aligned with MAFFT v7.123b in the local pair mode. Phylogenetic analysis was performed after removing all positions with gaps in the alignment; the maximum likelihood tree was then obtained with RAxML v8.2.10 using the GTRGAMMA model and 1000 bootstrap inferences. Moreover, pairwise distances between sequences were calculated using the Kimura model. In agreement with previous works, branch positions, pairwise distances, and collection countries were used to identify separate CCHFV clades within the phylogenetic tree [5,9,10,11,12,13,14,15,16,17,18,19,20,21,22].

### 2.3. Software Description

The homemade python script, named “CCHFV Primer Checker”, took the primers/probes listed in a table in the “csv” format, the multi-sequence alignment file in the “fasta” format, and, for each clade, the list of GeneBank accession numbers of all sequences to be analyzed, in the “txt” format. Both software and input files for the CCHFV analysis are posted in GitHub (https://github.com/cesaregruber/CCHFV-PrimerChecker).

As most heuristic alignment algorithms have difficulties in recovering the ends of the alignment region, when these contain mismatches [32], the “CCHFV Primer Checker” searched for primers and probe binding sites using a perfect-match approach with IUPAC ambiguity codes [33]. If the assay contained degenerate primers, the ambiguous characters were replaced by regular expressions (for example, the ambiguous character “N” is substituted by regular nucleotides A + C + G + T). The software found the start and end positions of the annealing sites of each primer and probes, with respect to IbAr10200 (NCBI reference sequence NC_005302).

The number and position of mismatches for every primer/probe with each CCHFV sequence was recorded by the program. The software calculated the percentage of mismatches between each primer/probe and all targets belonging to the same viral clade. The percentage of viral strains in each clade fully matching the primers and probe set (i.e., with no mismatch in the annealing sites) was also calculated.

The results were then displayed as an Excel file, reporting the frequency of mismatches between each primers/probe set and every genome sequence, for each clade. As it is known that a single 3’-end mismatch can affect the performance of the primers [26,27,32], all those “critical mismatches” occurring at the last five positions of the 3’-end primers were also recorded and reported.

To investigate the detection efficacy of a combination of assays, the software also evaluated the number of mismatches of all viral sequences in each clade with all possible assay combinations, with a maximum of five assays per combination.

### 2.4. Assays Evaluation

In order to guide the interpretation of the results of the in-silico analysis, for each viral clade three threshold parameters were assumed:

1. The primers/probe set of the assay must not have had more than 3 mismatches with respect to every genome in the clade;
2. The primers of the assay must not have had more than 1 critical mismatch, i.e., a mismatch located at the last 5 positions of 3’-end;
3. Within a clade, more than 50% of viral strains must have had fully matched the primers/probe set (therefore, we could expect that new sequences owing to the same clade would not have too many mismatches).

For each analyzed assay, the maximum number of mismatches of the respective primers/probe set, as well as that for each component of the set, were reported, together with the percent number of viral strains that fully matched each assay.

For each clade, the assay combination(s) with the minimum number of mismatches with all viral sequences was also reported.

## 3. Results

### 3.1. Data Collection

**Figure 1:**
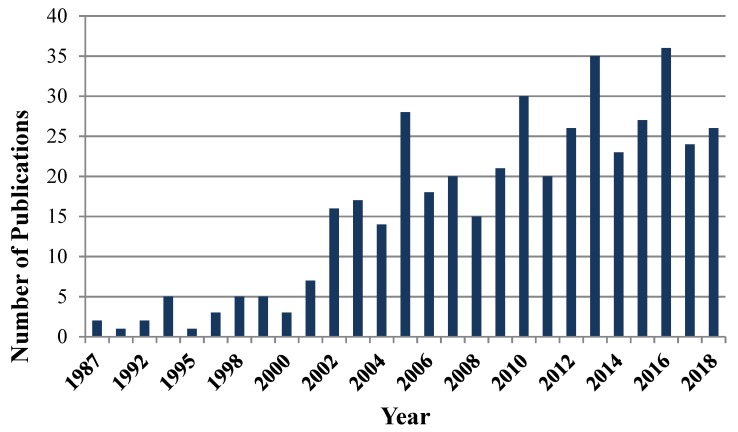
Publications on Crimean–Congo hemorrhagic fever virus (CCHFV). Frequency of CCHFV molecular diagnostics publications in English from 1992 to 2018.

The number of CCHFV molecular detection articles collected through PubMed search was 206 (Figure 1), most of them were discarded as they did not report detailed descriptions of the detection methods employed, including the sequences of primers and probes.

The complete list of published molecular assays published until December 2018 includes 2 single round PCR [34,35], 6 nested PCR [22,36,37,38,39], 12 real-time PCR [40,41,42,43,44,45,46,47,48,49,50,51], 1 LAMP [52]; and 1 RPA (Recombinase Polymerase Amplification) [53]. All assays are reported in Table 1 and in Supplementary Table S1. When available, reference testing materials and sensitivity/specificity of the methods are also reported in Table 1.

Starting with 2729 CCHFV sequences available in GenBank, 1,438 were found as the S segment, and 263 as complete coding sequences. The selection of records with available data about host, collection country and date, and clustering at 100% of nucleotide identity provided a total of 181 strains.

### 3.2. Phylogenetic Analysis

**Figure 2:**
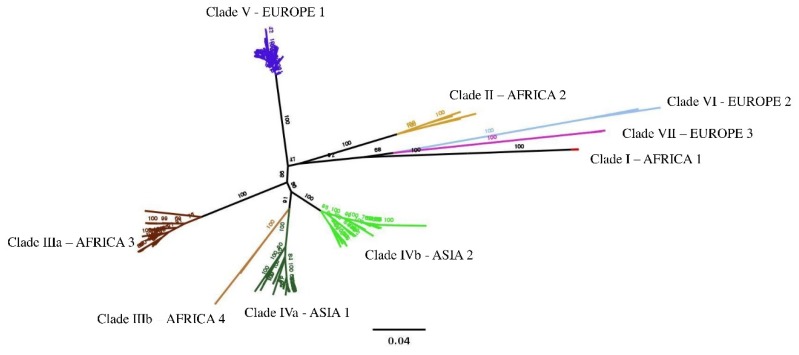
Maximum Likelihood Phylogenetic tree for complete S segment of CCHFV. Branches belonging to different clades are presented in different colors. Clade separation was adopted, in agreement with previous works (see Results), on the basis of collection countries and phylogeny. Asia 1: Oman, Iran Pakistan, Afghanistan, Kazakhstan, and United Arab Emirates; Asia 2: Iran, India, Tajikistan, Uzbekistan, Turkmenistan, China, and United Arab Emirates; Europe 2: Greece; Europe 3: Russia; Africa 3: Central and South Africa, and Spain; Africa 4: Nigeria and Spain.

The phylogenetic tree built with all 181 complete coding sequences of the S segment is shown in Figure 2. Our analysis suggests a clade separation into nine groups. In agreement with previous studies [5], we maintained the nomenclature of six different clades previously recognized, corresponding to different geographic regions—three clades prevalently diffused in Africa (Clades I–III), and two in Europe (Clades V and VI) and Asia (Clade IV). Moreover, one recently described clade was identified as clade VII [4] and, following previous work indications, clades III and IV were split into sub-groups, according to divergence in genetic distance (see web-only Supplementary Table S2) [17]. Median distance among CCHFV S sequences was 0.12 (range: 0.00–0.22), which was in agreement with [5]. The detailed phylogenetic tree is reported in the web-only Supplementary Figure S1.

### 3.3. Assays Evaluation

Using the CCHFV Primer Checker workflow, all sequence variants of CCHFV S segment versus the primers/ probe sets were identified for each molecular assay; reported in Supplementary Figure S2. The percentage of viral strains in each clade that fully matched (100% nucleotide identity) the set is reported in Table 2. For each viral clade, molecular assays that showed matching results over the thresholds values described in Methods, are reported in bold. As some molecular tests have an annealing site that lies in the extreme 5’ or 3’ non-coding regions, which is not represented in all of the analyzed S segments, the number of sequences included in the analysis varied between 89 and 160, according to the location of the primer/probe alignments. Therefore, for the assays falling in this category, we restricted the analysis to a reduced subset of CCHFV sequences. These results are presented in Table 3, since the value of this analysis is reduced and cannot be directly compared to the results reported for the other assays in Table 2.

As reported in Table 4, using a combination of assays resulted in a better match with sequences owing to all sub-groups, except Africa 1, Europe 2, and Europe 3. Assay combinations tested for all CCHFV sequences was also reported, using a maximum of five assays since the use of more than five tests is excessively demanding from a computational and a practical point of view. For all clades analyzed, combining more than three tests did not improve detection efficacy.

## 4. Discussion

An early and accurate diagnosis of CCHF infections is essential for case management and infection control procedures. The application of molecular tests in different settings is hampered by the great degree of sequence diversity. Therefore, serological methods have a broader use. The gold standard for diagnosis, however, is a combination of serology with molecular tests [4].

All available assays can be affected by the high diversity of the CCHFV genomes that hinders the design of specific primers/probe sets and prompts the use of high multiplexing procedures. Older molecular methods were designed on the basis of a limited number of CCHFV sequences and were often tested on a limited number of clades, while more recent assays were performed using bigger and updated data and were tested for multiple strain detection. Moreover, the methods optimized for the detection of strains circulating in a specific geographic area, might present a lower detection limit on the targeted strains, when compared with methods aimed to cover a broader spectrum of viral variants.

The in-silico PCR analysis performed in this study confirmed that assay sensitivity potential is strongly correlated to the geographic area of virus origin and to the evolutionary history of CCHFV, although validation studies with clinical samples are needed to proof this assumption. For example, the so-called “Drosten 2002” method perfectly matches more than 60% of sequences belonging to sub-group Asia 2 (Clade IV), but does not perfectly match any sequence belonging to other clades. Therefore, this assay can be considered useful to detect CCHFV from Asia but could perform worse when used to detect CCHFV from other regions. Our analysis confirmed suitability of primer choices for some assays that were expressly designed to detect CCHFV specifically from Europe, such as Duh 2006 (sub-group Europe 1) and Midilli 2009 (B) (sub-group Europe 2). A strong association between the predicted assay sensitivity and geographic origin emerged for other methods, like Wolfel 2007 (south-western Asia), Schneeberger 2017 (Central and Southern Africa, Spain, and Central Asia) and Jaaskelainen 2014 (Europe). Other assays seemed to be less affected by geographical variability, showing a good match with CCHFV strains belonging to different regions, such as Yapar 2005, Negredo 2017, Koheler 2018, and Sas 2018. In particular, even if specifically designed to detect Europe 2 sequences, Midilli 2007 (nested PCR) showed a good sequence homology with isolates from Asia, Africa, and European clades perfectly matching 75.9% to 84.8% of the considered CCHFV strains. For some assays (i.e., Deyde 2006, Atkinson 2012, and Bonney 2017), it is not feasible to perform a robust variant analysis using this tool because their target region includes the CCHFV 5’ end that is rarely sequenced and reported. Consequently, in silico evaluation on their diagnostic capacity are not supported by enough data. In particular, the assay from Deyde et al. (2006) covers both the 5’ and 3’ ends of the S segment and needs to be evaluated for potential sensitivity problems.

All parameters considered are intended as an easy guide for selecting the most appropriate assay for diagnostic purposes, even if further wet lab analysis on a wide panel of reference strains and real clinical samples are necessary to evaluate the sensitivity and specificity of molecular tests. However, limited reference strains are available for testing, even through specialized repositories like EVAg (www.european-virus-archive.com), and very few clinical samples are available for tuning the diagnostic capabilities. Interestingly, it could be noted that there were some agreements of our analysis with the EQA on the molecular detection of CCHFV [54]; in particular for Duh 2006, which was not able to detect strains from Asia 1 and Africa 3 clades. Therefore, in-silico evaluation, even if not free of drawbacks, still provides useful data for the choice of the most appropriate molecular method(s) to detect CCHFV from different endemic regions of the world. This analysis could not comprehend commercial assays (such as Altona), as they do not share primer sequences. We also emphasized the need for public sharing of primer design and selections for commercially offered assays, as part of capacity-building for emerging infections.

In conclusion, from this work emerges a strong region-dependence of potential performances of all assays, in detecting CCHFV. As more and more CCHFV sequences become available, our future efforts will be involved in the design of diagnostic tests capable to detect all known circulating CCHFV clades. Future molecular methods could be based on multiple tests, designed to detect multiple CCHFV targets at a high sensitivity and specificity. Potential design approaches based on Microarray or other High Multiplexing Fast PCR might be investigated. Currently, although a single method is to be considered appropriate for “local” investigations or in outbreak conditions, when the performance of the adopted method for the detection of the circulating strain is known, an effective diagnostic of CCHF in patients from different geographical areas should rely on a panel of methods and a thorough epidemiologic investigation.

## Figures and Tables

**Table 1:** Molecular assays for CCHFV detection published until 2018. For each assay, the type of molecular assay, the publication’s first author, the publication year, reference testing materials, and sensitivity/specificity of the tests are reported.

Assay Type	First Author	Year	Reference Testing Material	Declared Sensitivity/ Specificity
Single Round	Drosten	2002	Human clinical samples	LOD: 2779 copies/mL
	Deyde	2006	Human and animal laboratory isolates	N.D.
Nested	Schwarz	1996	Human serum samples	N.D.
	Midilli	2007	Human serum samples	N.D.
	Midilli (A)	2009	Human serum samples	N.D.
	Midilli (B)	2009	Human serum samples	N.D.
	Elata	2011	Human serum samples	N.D.
	Negredo	2017	Human serum samples	N.D.
Real Time	Yapar	2005	Human serum samples	LOD: 100 copies/mL
	Duh	2006	Human serum samples	LOD: 300 PFU/mL
	Garrison	2007	Laboratory isolates	LOD: 10 copies/mL
	Wolfel	2007	Human serum samples	LOD: 10 copies/mL
	Wolfel	2009	Laboratory isolates and human serum samples	LOD: 540 copies/mL
	Atkinson	2012	Laboratory isolates	LOD: 100 copies/mL
	Jaaskelainen	2014	Laboratory isolates and human serum samples	Sensitivity: N.D.; specificity: 97%
	Kamboj	2014	Animal	LOD: 7.6 copies (per reaction)
	Pang	2014	Laboratory isolates	LOD: 2000 copies/mL
	Koehler	2018	Laboratory isolates	LOD: 256 PFU/mL
	Sas	2018	Animals, humans and tick samples	LOD: 2000 copies/mL (gen II, IV, V and VI); 2 × 10^5^ copies/mL (gen III and I)
Sybr Green	Schneeberger	2017	Laboratory isolates	N.D.
LAMP	Osmann	2013	Human serum samples	LOD: ≥0.1 fg of viral RNA
RPA	Bonney	2017	Tick homogenates and clinical samples	LOD: between 500 and 50 copies (per reaction)

N.D.—not declared; LOD—limit of detection.

**Table 2:** Scoring summary of the molecular assays. For each clade the number of mismatches between the best primer/probe set and the most divergent sequence is reported. The apostrophes indicate the presence of one or more critical mismatches. The percentage of sequences with no mismatches are shown are shown in brackets. The best results (i.e., over all threshold parameters—see Methods) are shown in bold.

Type	Assay	Prim / Prob	Clade								
			Africa 1	Africa 2	Africa 3	Africa 4	Asia 1	Asia 2	Europe 1	Europe 2	Europe 3
			*N* = 2	*N* = 5	*N* = 29	*N* = 2	*N* = 30	*N* = 33	*N* = 76	*N* = 2	*N* = 2
Single Round	Drosten 2002	3	3 (0.0%)	5’ (0.0%)	4 (0.0%)	3 (0.0%)	4 (0.0%)	**2’ (60.6%)**	5 (0.0%)	9’ (0.0%)	4 (0.0%)
Nested	Schwarz 1996	4	5 (0.0%)	5’ (0.0%)	7’ (0.0%)	8’ (0.0%)	11’ (0.0%)	6’ (0.0%)	5 (0.0%)	13’’ (0.0%)	12’ (0.0%)
	Midilli 2007	3	5’ (0.0%)	8’’ (0.0%)	**2’ (75.9%)**	9’ (0.0%)	4’ (83.3%)	**1 (84.8%)**	**1 (84.2%)**	4’ (0.0%)	6’ (0.0%)
	Midilli 2009 (A)	4	16’’ (0.0%)	20’’ (0.0%)	21’’ (0.0%)	20’’ (0.0%)	21’’ (0.0%)	21’’ (0.0%)	21’’ (0.0%)	4’ (50.0%)	16’’ (0.0%)
	Midilli 2009 (B)	4	11’’ (0.0%)	10’’ (0.0%)	14’’ (0.0%)	8’’ (0.0%)	12’’ (0.0%)	11’’ (0.0%)	4’ (71.1%)	12’ (0.0%)	10’’ (0.0%)
	Elata 2011	4	11’’ (0.0%)	10’ (0.0%)	8’ (62.1%)	12’ (0.0%)	11’ (0.0%)	11’ (0.0%)	10’’ (0.0%)	14’’ (0.0%)	14’’ (0.0%)
	Negredo 2017	4	**0 (100%)**	4’’ (20.0%)	**1 (89.7%)**	4 (0.0%)*	3’’ (80.0%)	**2’ (87.9%)**	**1 (86.8%)**	**0 (100%)**	4 (0.0%)
Real Time	Yapar 2005	2 / 1	**0 (100%)**	1 (40.0%)	2 (0.0%)	2 (0.0%)	4’ (0.0%)	**1 (60.6%)**	**1 (93.4%)**	2 (0.0%)	1 (0.0%)
	Duh 2006	2 / 1	11’ (0.0%)	7’ (0.0%)	9’ (0.0%)	5’’ (0.0%)	8’ (0.0%)	8’ (0.0%)	3’ (25.0%)	9’ (0.0%)	7’ (0.0%)
	Garrison 2007	2 / 1	10’ (0.0%)	4 (0.0%)	9’’ (0.0%)	7’ (0.0%)	9’’ (0.0%)	5’ (12.1%)	7’ (0.0%)	11’’ (0.0%)	5’ (0.0%)
	Wolfel 2007	2 / 2	8’ (0.0%)	6 (0.0%)	7’ (0.0%)	4 (0.0%)	3 (0.0%)	4’ (9.1%)	6’ (0.0%)	6’ (0.0%)	9 (0.0%)
	Wolfel 2009	6 / 13	7 (0.0%)	8 (0.0%)	6’’ (24.1%)	2 (0.0%)	3’’ (73.3%)	5’’ (0.0%)	5’ (0.0%)	8’’ (0.0%)	5 (0.0%)
	Jaaskelainen 2014	3 / 3	4’’ (0.0%)	6 (0.0%)	4 (37.9%)	6 (0.0%)	7’ (0.0%)	7’ (0.0%)	**3’ (81.6%)**	4’’ (0.0%)	6 (0.0%)
	Pang 2014	2 / 1	9’ (0.0%)	11 (0.0%)	3 (3.4%)	4’ (0.0%)	3’ (0.0%)	5’’ (0.0%)	4’ (14.5%)	12 (0.0%)	12’ (0.0%)
	Koehler 2018	2 / 1	2 (0.0%)	**2 (80.0%)**	**3’ (72.4%)**	3 (0.0%)	4’’ (0.0%)	3 (24.2%)	**2 (85.5%)**	5’’ (0.0%)	4’ (0.0%)
	Sas 2018	14 / 2	**0 (100%)**	3 (0.0%)	3’ (37.9%)	2 (0.0%)	**3 (73.3%)**	**3’ (51.5%)**	**2 (82.9%)**	2 (50.0%)	7 (0.0%)
Sybr Green	Schneeberger 2017	2	6’ (0.0%)	6’ (0.0%)	3’ (17.2%)	3 (0.0%) *	5’ (0.0%)	2’’ (27.3%)	7’ (0.0%)	9’ (0.0%)	4 (0.0%)
LAMP	Osmann 2013	8	24’’ (0.0%)	23’ (0.0%)	8’’ (6.9%)	19’ (0.0%)	19’’ (0.0%)	23’’ (0.0%)	21’’ (0.0%)	28’’ (0.0%)	25’ (0.0%)

* Spain sequence (Africa 4) not available at forward primer binding site; ’ One mismatch found at the last 5 nt of 3’ primers; ’’ Two or more mismatches found at the last 5 nt of 3’ primers; N: number of sequences used as target.

**Table 3:** Scoring summary of the molecular assays with an annealing site lying in the extreme 5’ or 3’ non-coding regions.

Type	Assay	Number of Primers/Probes	Clade								
			Africa 1	Africa 2	Africa 3	Africa 4	Asia 1	Asia 2	Europe 1	Europe 2	Europe 3
			*N* = 2	*N* = 1	*N* = 15	*N* = 0	*N* = 16	*N* = 20	*N* = 33	*N* = 2	*N* = 0
PCR	Deyde 2006	2 primers	0 (100.0%)	0 (100.0%)	1 (93.3%)	- (-)	9 (87.5%)	2’’ (90.0%)	1 (90.9%)	0 (100.0%)	- (-)
			*N* = 2	*N* = 2	*N* = 21	*N* = 0	*N* = 17	*N* = 24	*N* = 33	*N* = 2	*N* = 0
RealTime	Atkinson 2012	2 primers / 1 probe	3 (0.0%)	6 (0.0%)	3 (0.0%)	- (-)	15’ (0.0%)	6 (0.0%)	5 (0.0%)	5 (0.0%)	- (-)
			*N* = 2	*N* = 4	*N* = 27	*N* = 1	*N* = 21	*N* = 29	*N* = 72	*N* = 2	*N* = 2
RealTime	Kamboj 2014	2 primers / 1 probe	7’ (0.0%)	12’’ (0.0%)	5’’ (0.0%)	7’’ (0.0%)	8’’ (0.0%)	3’’ (24.1%)	9’’ (0.0%)	8’’ (0.0%)	15’’ (0.0%)
			*N* = 2	*N* = 2	*N* = 22	*N* = 0	*N* = 19	*N* = 24	*N* = 58	*N* = 2	*N* = 0
RPA	Bonney 2017	2 primers / 1 probe	9’ (0.0%)	11 (0.0%)	8’ (0.0%)	- (-)	10 (0.0%)	9 (0.0%)	5 (46.6%)	11 (0.0%)	- (-)

’ One mismatch found at the last 5 nt of 3’ primers; ’’ Two or more mismatches found at the last 5 nt of 3’ primers; N: number of sequences used as target.

**Table 4:** Best assay combinations for the CCHFV detection. For each clade, the combination(s) of assays with the best detection efficacy was reported on the basis of the three threshold parameters (see Methods).

Clade		Best Assay Combination	Equivalent Combination	Max Mismatches Per Seq	Max Mismatches in Last 5 nt	Perfect Matched Sequences
Africa 1	*N* = 2	Negredo 2017	Yapar 2005 OR Sas 2018	0	0	100.0%
Africa 2	*N* = 5	Yapar 2005 + Koehler 2018		1	0	80.0%
Africa 3	*N* = 29	Elata 2011 + Negredo 2017		0	0	100.0%
Africa 4	*N* = 2	Sas 2018 + Yapar 2005	Yapar 2005 + Drosten 2002	1	0	0.0%
Asia 1	*N* = 30	Midilli 2007 + Wolfel 2009 + Koehler 2018		1	0	90.0%
Asia 2	*N* = 33	Midilli 2007 + Schneeberger 2017 + Drosten 2002	Midilli 2007 + Schneeberger 2017 + Kamboj 2014 OR Negredo 2017 + Schneeberger 2017 + Kamboj 2014 OR Negredo 2017 + Schneeberger 2017 + Drosten 2002	0	0	100.0%
Europe 1	*N* = 76	Yapar 2005 + Midilli 2007	Yapar 2005 + Negredo 2017	0	0	100.0%
Europe 2	*N* = 2	Negredo 2017		0	0	100.0%
Europe 3	*N* = 2	Yapar 2005		1	0	0.0%
All	*N* = 181	Yapar 2005 + Negredo 2017 + Koehler 2018 + Drosten 2002 + Wolfel 2009		1	0	93.4%

## Supplementary Materials

The following are available online at https://www.mdpi.com/1999-4915/11/10/953/s1, Figure S1: Detailed phylogenetic tree. Figure S2: Sequence variants of the CCHFV S segment versus each molecular assay. Table S1: Published molecular assays for CCHFV detection at December 2018. Table S2: CCHFV genetic distances between all analyzed CCHFV genomes, calculated using the Kimura-2-parameters model.
