# Supplementary material for: Geographical Variability Affects CCHFV Detection by RT–PCR: A Tool for In-Silico Evaluation of Molecular Assays
Source: Viruses. 2019 Oct 16;11(10):953. doi: 10.3390/v11100953 (PMC6833031; doi:10.3390/v11100953)
Supplement: Supplementary file 1 [file viruses-11-00953-s001.zip › viruses-623856 final supplementary/SupplementaryFiles-New/FigureS2-Primers.pdf]

Single Round PCR

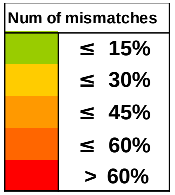

Drosten et al. 2002

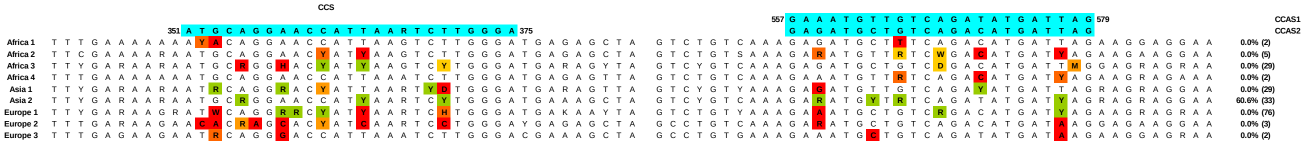

Deyde et al. 2006

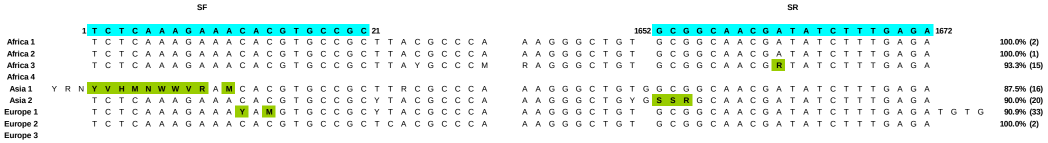

RPA

Bonney et al. 2017

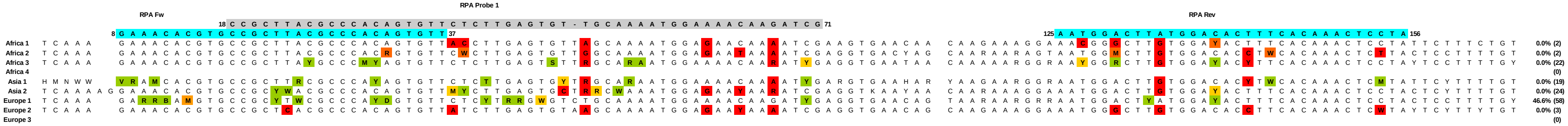

LAMP

Osmann et al. 2013

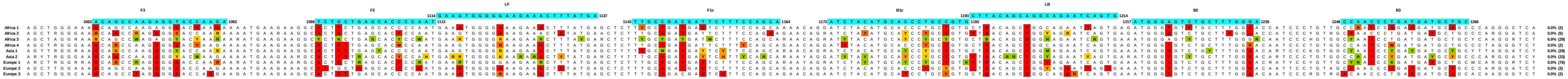

Nested

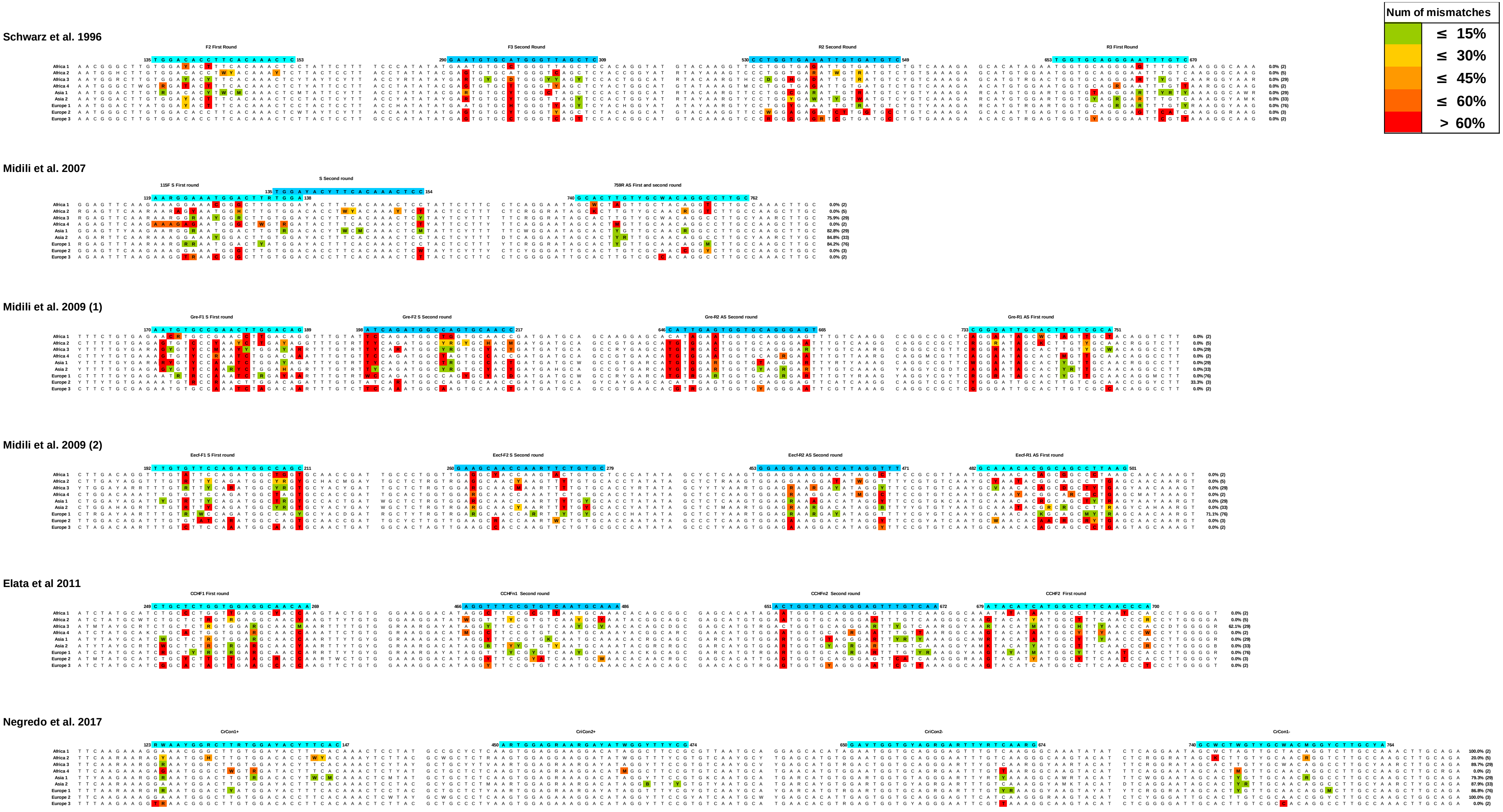
Num of mismatches
≤ 15%
≤ 30%
≤ 45%
≤ 60%
> 60%
Schwarz et al. 1996
F2 First Round
F3 Second Round
R2 Second Round
R3 First Round
Midili et al. 2007
115F S First round
S Second round
759R AS First and second round
Midili et al. 2009 (1)
Gre-F1 S First round
Gre-F2 S Second round
Gre-R2 AS Second round
Gre-R1 AS First round
Midili et al. 2009 (2)
Eecf-F1 S First round
Eecf-F2 S Second round
Eecf-R2 AS Second round
Eecf-R1 AS First round
Elata et al 2011
CCHF1 First round
CCHFn1 Second round
CCHFn2 Second round
CCHF2 First round
Negredo et al. 2017
CrCon1+
CrCon2+
CrCon2-
CrCon1-
Africa 1
Africa 2
Africa 3
Africa 4
Asia 1
Asia 2
Europe 1
Europe 2
Europe 3

Real Time

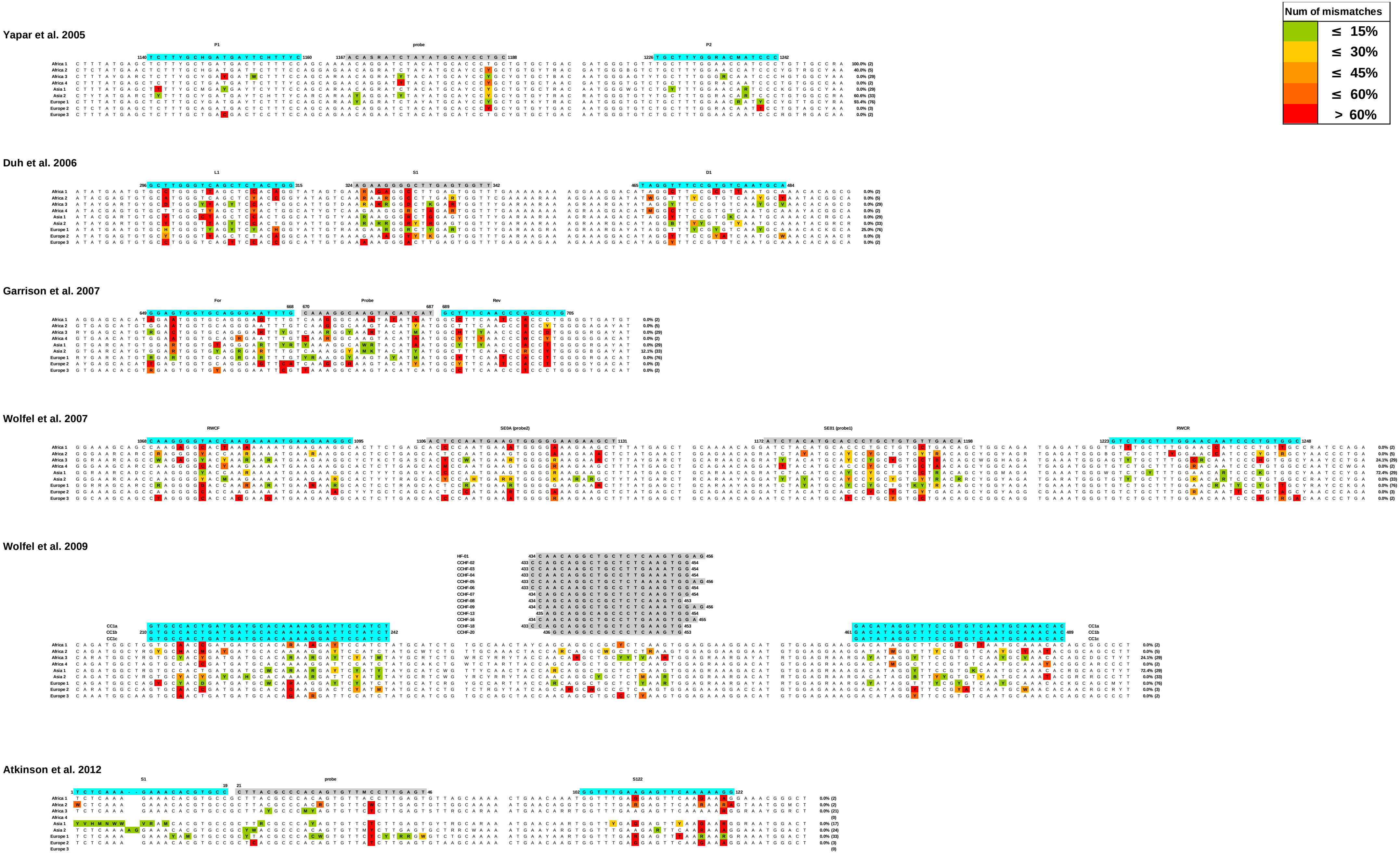

Num of mismatches
≤ 15%
≤ 30%
≤ 45%
≤ 60%
> 60%
Yapar et al. 2005
P1
probe
P2
Duh et al. 2006
L1
S1
D1
Garrison et al. 2007
For
Probe
Rev
Wolfel et al. 2007
RWCF
SE0A (probe2)
SE01 (probe1)
RWCR
Wolfel et al. 2009
CC1a
CC1b
CC1c
Atkinson et al. 2012
S1
probe
S122
Africa 1
Africa 2
Africa 3
Africa 4
Asia 1
Asia 2
Europe 1
Europe 2
Europe 3

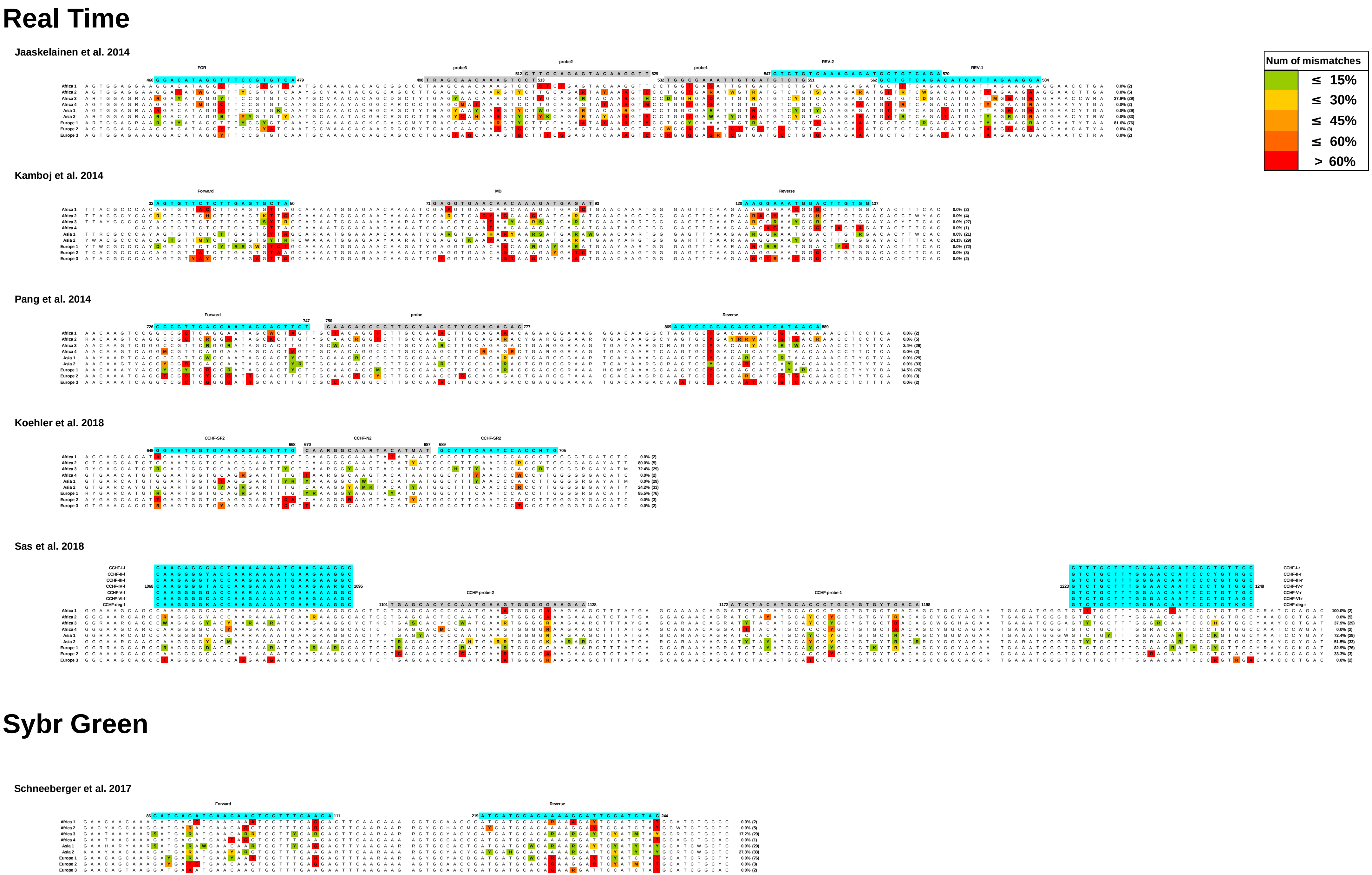

Real Time
Jaaskelainen et al. 2014
FOR
probe3
probe2
probe1
REV-2
REV-1
Kamboj et al. 2014
Forward
MB
Reverse
Pang et al. 2014
Forward
probe
Reverse
Koehler et al. 2018
CCHF-SF2
CCHF-N2
CCHF-SR2
Sas et al. 2018
CCHF-probe-2
CCHF-probe-1
Sybr Green
Schneeberger et al. 2017
Forward
Reverse
Africa 1
Africa 2
Africa 3
Africa 4
Asia 1
Asia 2
Europe 1
Europe 2
Europe 3
Num of mismatches
≤ 15%
≤ 30%
≤ 45%
≤ 60%
> 60%
