# Supplementary material for: Geographical Variability Affects CCHFV Detection by RT–PCR: A Tool for In-Silico Evaluation of Molecular Assays
Source: Viruses. 2019 Oct 16;11(10):953. doi: 10.3390/v11100953 (PMC6833031; doi:10.3390/v11100953)
Supplement: Supplementary file 1 [file viruses-11-00953-s001.zip › viruses-623856 final supplementary/SupplementaryFiles-New/Table S1.pdf]

Supplementary Table S1. Published molecular assays for Crimean-Congo haemorrhagic fever virus detection as at December 2018.

Assay	Reference	Reference testing material	Declared sensitivity/specificity	Position in CCHFV strain IbAr10200	Primers and probes		
					Type	Name	Sequence
Single round PCR	Drosten 2002	Human clinical samples	95% detection limit of 2,779 copies per mL of serum	351–579	Forward primer	CCS	ATGCAGGAACCATTAARTCTTGGGA
					Reverse primer	CCAS1	CTAATCATATCTGACAACATTTC
					Additional reverse primer	CCAS2	CTAATCATGTCTGACAGCATCTC
	Deyde 2006	Human and animal laboratory isolates	ND	1–1,672	Forward primer	SF	TCTCAAAGAAACACGTGCCGC
					Reverse primer	SR	TCTCAAAGATATCGTTGCCGC
Nested PCR	Schwarz 1996	Human serum samples	ND	135–670	Forward out	F2	TGGACACCTTCACAAACTC
					Reverse out	R2	GACATCACAATTTCACCAGG
					Forward inn	F3	GAATGTGCATGGGTTAGCTC
					Reverse inn	R3	GACAAATTCCCTGCACCA
	Midili 2007	Human serum samples	ND	119–762	Forward out	CCF-115F	AARGGAAATGGACTTRTGGA
					Forward inn	CCF-131F	TGGAYACYTTCACAAACTCC

CCHFV: Crimean Congo haemorrhagic fever virus; LAMP: loop-mediated isothermal amplification; ND: not declared; PFU: Plaque Forming Units; RPA: recombinase polymerase amplification; RT: reverse transcription. For each assay, the position of amplicon is reported to respect IbAr10200 (NCBI reference sequence NC_005302). Names of each primer and probe correspond to those reported in the reference.

Supplementary Table S1. Published molecular assays for Crimean-Congo haemorrhagic fever virus detection as at December 2018.

					Reverse out/inn	CCF-759R	GCAAGGCCTGTWGCRACAAGTGC
Nested PCR	Midili 2009 (1)	Human serum samples	ND	170–751	Forward out	Gre-F1	AATGTGCCGAACTTGGACAG
					Reverse out	Gre-R1	TGCGACAAGTGCAATCCCG
					Forward inn	Gre-F2	ATCAGATGGCCAGTGCAACC
					Reverse inn	Gre-R2	ACTCCCTGCACCACTCAATG
	Midili 2009 (2)	Human serum samples	ND	192–501	Forward out	Eecf-F1	TTGTGTTCCAGATGGCCAGC
					Reverse out	Eecf-R1	CTTAAGGCTGCCGTGTTTGC
					Forward inn	Eecf-F2	GAAGCAACCAARTTCTGTGC
					Reverse inn	Eecf-R2	AAACCTATGTCCTTCCTCC
	Elata 2011	Human serum samples	ND	249–700	Forward out	CCHF1	CTGCTCTGGTGGAGGCAACAA
					Reverse out	CCHF2_5	TGGGTTGAAGGCCATGATGTAT
					Forward inn	CCHFn15	AGGTTTCCGTGTCAATGCAAA
					Reverse inn	CCHFn25	TTGACAAACTCCCTGCACCAGT
	Negredo 2017	Human serum samples	ND	123–764	Forward out	CrCon1 +	RWAAYGGRCTTRTGGAYACYTTCAC
						CrCon1-	TRGCAAGRCCKGTWGCRACWAGWGC

CCHFV: Crimean Congo haemorrhagic fever virus; LAMP: loop-mediated isothermal amplification; ND: not declared; PFU: Plaque Forming Units; RPA: recombinase polymerase amplification; RT: reverse transcription. For each assay, the position of amplicon is reported to respect IbAr10200 (NCBI reference sequence NC_005302). Names of each primer and probe correspond to those reported in the reference.

Supplementary Table S1. Published molecular assays for Crimean-Congo haemorrhagic fever virus detection as at December 2018.

					Reverse out		
					Forward inn	CriCon2 +	ARTGGAGRAARGAYATWGGYTTYCG
Nested PCR	Negredo 2017				Reverse inn	CriCon2-	CYTTGAYRAAYTCYCTRCACCABTC
Real-time PCR	Yapar 2005	Human serum samples	Linear detection 10^7–10^2 copies/mL	1,140–1,242	Forward primer	CCRealP1	TCTTYGCHGATGAYTCHTTYC
					Reverse primer	CCRealP2	GGGATKGTYCCRAAGCA
					Probe	ND	ACASRATCTAYATGCAYCCTGC
	Duh 2006	Human serum samples	Viral RNA was detected until 30 PFU/mL	296–484	Forward primer	CCHFL1	GCTTGGGTCAGCTCTACTGG
					Reverse primer	CCHFD1	TGCATTGACACGGAAACCTA
					Probe	CCHFS1	AGAAGGGGCTTGAGTGGTT
	Wolfel 2007	Human serum samples	Analytical sensitivity in concentrations ranging from 100,000 –10 copies per mL	1,068–1,248	Forward primer	RWCF	CAAGGGGTACCAAGAAAATGAAGAAGGC
					Reverse primer	RWCR	GCCACAGGGATTGTTCCAAAGCAGAC
					Probe	SE01	ATCTACATGCACCCTGCTGTGTTGACA
					Additional probe	SE03	ATTTACATGCACCCTGCCGTGCTTACA
					Additional probe	SE0A	AGCTTCTTCCCCCACTTCATTGGAGT
	Garrison 2007	Laboratory isolates	Limit of detection 10 copies/mL; from 1.18 x 10^6 – 11.8 gene copies were linear	649–705	Forward primer	CCHF	GGAGTGGTGCAGGGAATTTG
					Reverse primer	CCHF	CAGGGCGGGTTGAAAGC

CCHFV: Crimean Congo haemorrhagic fever virus; LAMP: loop-mediated isothermal amplification; ND: not declared; PFU: Plaque Forming Units; RPA: recombinase polymerase amplification; RT: reverse transcription. For each assay, the position of amplicon is reported to respect IbAr10200 (NCBI reference sequence NC_005302). Names of each primer and probe correspond to those reported in the reference.

Supplementary Table S1. Published molecular assays for Crimean-Congo haemorrhagic fever virus detection as at December 2018.

					Probe	CCHF	CAAAGGCAAGTACATCAT
Real-time PCR	Wolfel 2009	Laboratory isolates and human serum samples	95% detection limit of 540 copies/mL of serum, corresponding to 6.3 genome copies/reaction	210–489	Forward primer	CC1a_for	GTGCCACTGATGATGCACAAAAGGATTCCATCT
					Reverse primer	CC1a_rev	GTGTTTGCATTGACACGGAAACCTATGTC
					Probe	HF-01	CAACAGGCTGCTCTCAAGTGGAG
					Additional forward primer	CC1b_for	GTGCCACTGATGATGCACAAAAGGATTCTATCT
						CC1c_for	GTGCCACTGATGATGCACAAAAGGACTCCATCT
					Additional reverse primer	CC1b_rev	GTGTTTGCATTGACACGGAAGCCTATGTC
						CC1c_rev	GTGTTTGCATTGACACGGAAACCTATATC
					Additional probe	CCHF-02	CAACAGGCTGCTCTCAAGTGGAG
						CCHF-03	CCAGCAGGCTGCTCTCAAGTGG
						CCHF-04	CCAACAAGCTGCCTTGAAATGG
						CCHF-05	CCAACAGGCTGCCTTGAAATGG
						CCHF-06	CCAACAGGCTGCTCTAAAGTGGAG
						CCHF-07	CCAACAAGCTGCCTTGAAGTGG
						CCHF-08	CAGCAGGCTGCTCTCAAGTGG
						CCHF-09	CAGCAGGCCGCTCTCAAGTG
						CCHF-13	CAACAGGCTGCTCTCAAATGGAG
						CCHF-16	AGCAGGCAGCCCTCAAGTGG
						CCHF-18	CAACAGGCTGCCTTGAAGTGGA
						CCHF-20	CCAGCAGGCTGCTCTGAAGTG

CCHFV: Crimean Congo haemorrhagic fever virus; LAMP: loop-mediated isothermal amplification; ND: not declared; PFU: Plaque Forming Units; RPA: recombinase polymerase amplification; RT: reverse transcription. For each assay, the position of amplicon is reported to respect IbAr10200 (NCBI reference sequence NC_005302). Names of each primer and probe correspond to those reported in the reference.

Supplementary Table S1. Published molecular assays for Crimean-Congo haemorrhagic fever virus detection as at December 2018.

Real-time PCR	Atkinson 2012	Laboratory isolates	Ranging from $5x10^5$, down to 0.5 copies of S segment RNA per reaction	1–122	Forward primer	CCHFS1	TCTCAAAGAAACACGTGCC
					Reverse primer	CCHFS122	CCTTTTTGAACTCTTCAAACC
					Probe	ND	ACTCAAGGKAACACTGTGGGCGTAAG
	Jaaskelainen 2014	Laboratory isolates and human serum samples	Sensitivity 100%;specificity 97%	460–584	Forward primer	FOR	GGACATAGGTTTCCGTGTCA
					Reverse primer	REV-1	TCCTTCTAATCATGTCTGACAGC
					Additional reverse primer	REV-2	TCTGACAGCATCTCTTTGACAGAC
					Probe	probe1	TGGCGAAATTGTGATGTCTG
					Additional probe	probe2	CTTGCAGAGTACAAGGTT
					Additional probe	probe3	TRAGCAACAAAGTCCT
	Kamboj 2014	Animal	Sensitivity from 7.6 x 10^9 –7.6 copies:specificity 100%	32–137	Forward primer	ND	AGTGTTCTCTTGAGTGCTA
					Reverse primer	ND	CCACAAGTCCATTTCCTT
					Probe	ND	ATCTCATCTTTGTTGTTCACCTC
	Pang 2014	Laboratory isolates	Limit of detection from 133 RNA copies/PCR	726–889	Forward primer	ND	GCCGTTCAGGAATAGCACTTGT
					Reverse primer	ND	TGTTATCATGCTGTCGGCRCT
					Probe	ND	CAACAGGCCTTGCYAAGCTYGCAGAGAC
	Koehler 2018	Laboratory isolates	Limit of detection: 256 FU/ml	649-705	Forward primer	CCHF-SF2	GGAVTGGTGVAGGGARTTTG

CCHFV: Crimean Congo haemorrhagic fever virus; LAMP: loop-mediated isothermal amplification; ND: not declared; PFU: Plaque Forming Units; RPA: recombinase polymerase amplification; RT: reverse transcription. For each assay, the position of amplicon is reported to respect IbAr10200 (NCBI reference sequence NC_005302). Names of each primer and probe correspond to those reported in the reference.

Supplementary Table S1. Published molecular assays for Crimean-Congo haemorrhagic fever virus detection as at December 2018.

					Reverse primer	CCHF-SR2	CADGGTGGRTTGAARGC
					Probe	CCHF-N2	6CAARGGCAARTACATMAT
	Sas 2018	Animals, humans and ticks samples	Limit of detection: VI 2 copies/µl (genotypes II, IV, V); 200 copies/µl (genotypes III and I).	1068-1248	Forward primer	CCHF-I-f	CAAGAGGCACTAAAAAAATGAAGAAGGC
						CCHF-II-f	CAAGGGGYACCAARAAAATGAAGAAGGC
						CCHF-III-f	CAAGAGGTACCAAGAAAATGAAGAAGGC
						CCHF-IV-f	CAAGGGGTACCAAGAAAATGAAGAARGC
						CCHF-V-f	CAAGGGGGACCAARAAAATGAAAAAGGC
						CCHF-VI-f	CAAGGGGCACCAAGAAAATGAAGAAAGC
						CCHF-deg-f	CAAGGGGKACCAAGAAAATGAARAAGGC
					Reverse primer	CCHF-I-r	GCAACAGGGATGGTTCCAAAGCAAAC
						CCHF-II-r	GCYACRGGGATGGTTCCRAAGCAGAC
						CCHF-III-r	GCCACGGGGATTGTCCCAAAGCAGAC
						CCHF-IV-r	GCCACAGGGATTGTTCCAAAGCAGAC
						CCHF-V-r	GCAACAGGGATTGTTCCAAAGCAGAC
						CCHF-VI-r	GCTACAGGAATTGTCCCAAAGCAGAC
						CCHF-deg-r	GCMACAGGGATTGTYCCAAAGCAGAC
					Probe	CCHF-probe-1	ATCTACATGCACCCTGCYGTGYTGACA
Real-time PCR	Sas 2018					CCHF-probe-2	TTCTTCCCCCACTTCATTGGRGTGCTCA
Sybrgreen Real Time	Schneeberger 2017	Laboratory isolates	ND	86–244	Forward primer	ND	GATGAGATGAACAAGTGGTTTGAAGA
					Reverse primer	ND	GTAGATGGAATCCTTTTGTGCATCAT

CCHFV: Crimean Congo haemorrhagic fever virus; LAMP: loop-mediated isothermal amplification; ND: not declared; PFU: Plaque Forming Units; RPA: recombinase polymerase amplification; RT: reverse transcription. For each assay, the position of amplicon is reported to respect IbAr10200 (NCBI reference sequence NC_005302). Names of each primer and probe correspond to those reported in the reference.

Supplementary Table S1. Published molecular assays for Crimean-Congo haemorrhagic fever virus detection as at December 2018.

LAMP	Osman 2013	Human serum samples	Sensitivity in detecting ≥ 0.1 fg of viral RNA 100%	1,063–1,266	Forward outer primer	F3	ACAGCCAAGAGGTACCAAGA
					Reverse outer primer	B3	GCAGCATCATCAGGGTTGG
					Inner primers	F1c	TCTGCTGAGCACCCCAAT
						F2	TGCTGGAAAGAATCGTCGGCAA
						B2	TCCCAAAGCAGACTCCCAT
						B1c	ATCTACATGCACCCTGCCGTG
					Loop primers	LF	TCATAAAGTTTCTTCCCCCACTTC
LAMP	Osman 2013					LB	CTTACAGCAGGCAGAATCAGTG
RPA	Bonney 2017	Laboratory isolates	Limit of detection from $5x10^6$ template copies to 50 copies	8–158	Forward primer	ND	GAAACACGTGCCGCTTACGCCCACAGTGTT
					Reverse primer	ND	TAGGAGTTTGTGAAAGTGTCCATAAGTCCATT
					Probe	ND	CCGCTTACGCCCACAGTGTTCTCTTGAGTGTNTGCAAAATGGAAAACAAGATCG

CCHFV: Crimean Congo haemorrhagic fever virus; LAMP: loop-mediated isothermal amplification; ND: not declared; PFU: Plaque Forming Units; RPA: recombinase polymerase amplification; RT: reverse transcription. For each assay, the position of amplicon is reported to respect IbAr10200 (NCBI reference sequence NC_005302). Names of each primer and probe correspond to those reported in the reference.
