# Supplementary figures and images for: Geographical Variability Affects CCHFV Detection by RT–PCR: A Tool for In-Silico Evaluation of Molecular Assays
Source: Viruses. 2019 Oct 16;11(10):953. doi: 10.3390/v11100953 (PMC6833031; doi:10.3390/v11100953)

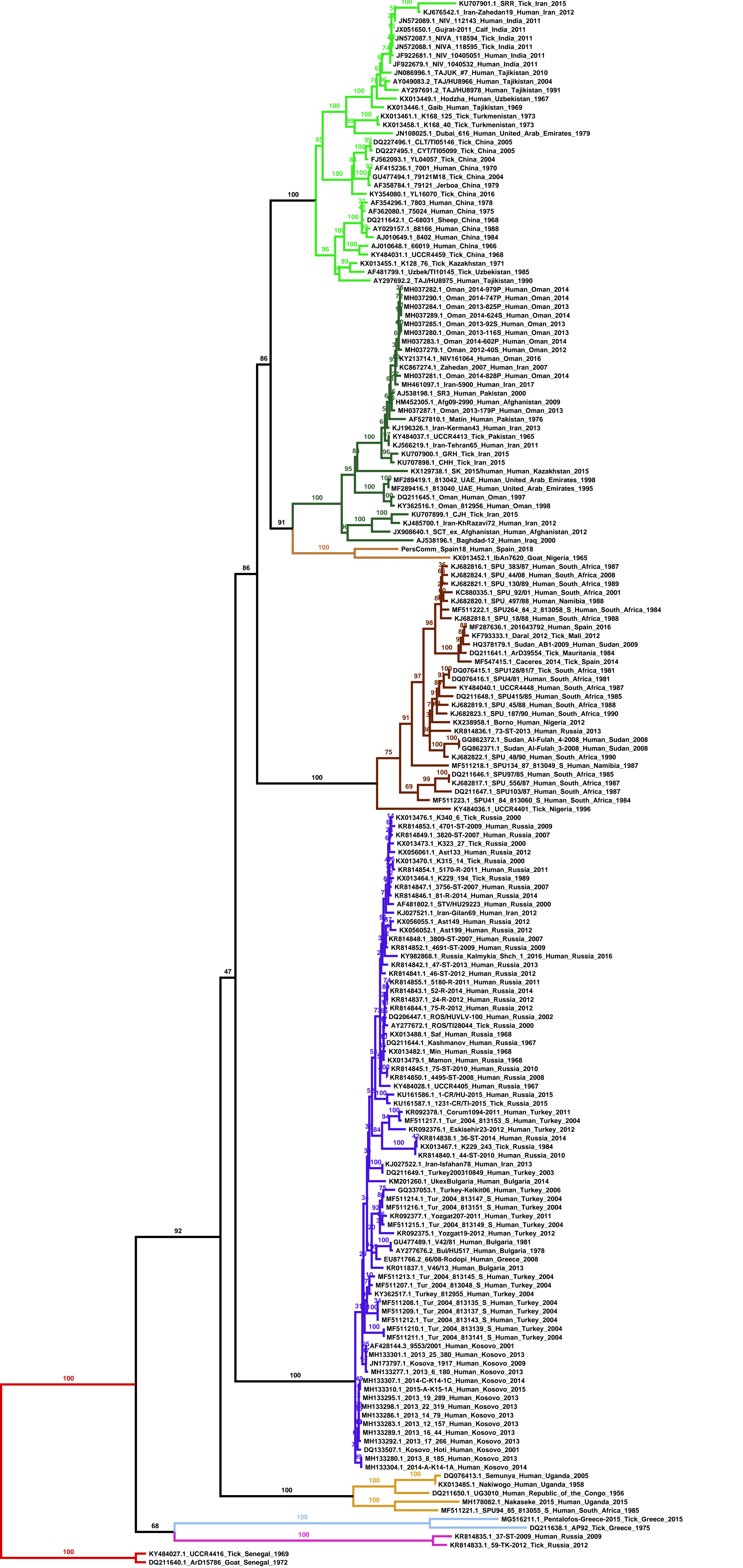

Supplement: Supplementary file 1 [file viruses-11-00953-s001.zip › viruses-623856 final supplementary/SupplementaryFiles-New/FigureS1-ExpandedPhylogeneticTree.pdf]
